# Delivery of a Mental Health First Aid training package and staff peer support service in secondary schools: a process evaluation of uptake and fidelity of the WISE intervention

**DOI:** 10.1186/s13063-020-04682-8

**Published:** 2020-08-26

**Authors:** Harriet Fisher, Sarah Harding, Sarah Bell, Lauren Copeland, Rhiannon Evans, Jillian Powell, Ricardo Araya, Rona Campbell, Tamsin Ford, David Gunnell, Simon Murphy, Judi Kidger

**Affiliations:** 1 Population Health Sciences, Bristol Medical School, University of Bristol, Canynge Hall, 39 Whatley Road, Bristol, BS8 2PS UK; 2 School of Social Sciences, Cardiff University, Cardiff, UK; 3 Health Service and Population Research Department, Kings College London, London, UK; 4 College of Medicine and Health, University of Exeter, Exeter, UK

**Keywords:** Process evaluation, Teachers, Schools, Mental health, Wellbeing

## Abstract

**Background:**

Improving children and young people’s provision for mental health is a current health priority in England. Secondary school teachers have worse mental health outcomes than the general working population, which the Wellbeing in Secondary Education (WISE) cluster randomised controlled trial aimed to improve. The WISE intervention comprised a Mental Health First Aid (MHFA) training package delivered to at least 16% of staff, a short mental health awareness session to all teachers and development of a staff peer support service. Twenty-five schools were randomised to intervention or control arms. This paper reports findings regarding the extent of uptake and fidelity of the intervention.

**Methods:**

Mixed methods data collection comprised researcher observations of training delivery, training participant evaluation forms, trainer and peer supporter interviews, peer supporter feedback meetings, logs of support provided, and teacher questionnaires. Quantitative data were summarised descriptively, while thematic analysis was applied to the qualitative data.

**Results:**

In the 12 schools assigned to the intervention arm, 113 (8.6%) staff completed the 2-day standard MHFA training course, and a further 146 (11.1%) staff completed the 1-day MHFA for schools and colleges training. In seven (58.3%) schools, the required 8% of staff completed the MHFA training packages. A 1-h mental health awareness-raising session was attended by 666 (54.5%) staff. Delivery of the MHFA training package was achieved with high levels of fidelity and quality across schools. All schools set up the peer support service following training, with a majority adhering to most of the operational guidelines developed from the pilot study at the outset. Teachers reported limited use of the peer support service during follow-up. At the 1-year follow-up, only three (25.0%) schools indicated they had re-advertised the service and there was evidence of a reduction in support from senior leadership.

**Conclusion:**

The MHFA training package was delivered with reasonably high fidelity, and a staff peer support service was established with general, but not complete, adherence to guidelines. In some schools, insufficient staff received MHFA training and levels of delivery of the peer support service compromised intervention dose and reach.

**Trial registration:**

ISRCTN 95909211. Registered on 15 January 2016

## Contributions to the literature

- Improving teachers’ mental wellbeing is a public health concern which the WISE cluster randomised controlled trial aimed to improve
- The embedded process evaluation showed uptake of a Mental Health First Aid training package in secondary schools can be achieved with reasonably high levels of fidelity and quality
- A staff peer support service can be established in schools, but challenges to ongoing delivery compromised intervention dose and reach

## Background

Secondary school teachers have poorer emotional wellbeing, higher prevalence of depressive symptoms, and increased levels of stress and anxiety-related disorders compared to the general population [1, 2]. Interventions to improve the mental health of secondary school teachers are important to reduce the risk of them developing more serious, longer-term mental health conditions [3], and negate the adverse impact on teacher–student relationships and learning outcomes of students that may accompany poor mental health in their teachers [4, 5]. Improvements to public mental health literacy may lead to better mental health outcomes, by facilitating early help-seeking behaviours, by equipping others to identify signs of mental health difficulties earlier [6] and by reducing the stigma associated with mental illness.

With the aim of improving provision for children and young people’s mental health, the UK government have recently pledged Mental Health First Aid (MHFA) training for a single staff member at each secondary school in England [7]. MHFA is an internationally recognised training course designed to teach lay people first aid skills to support others with mental health problems [8]. The training aims to teach individuals practical skills that can be used to identify signs and symptoms of mental health difficulties, and provide confidence in guiding people towards appropriate support [9]. Youth Mental Health First Aid training has been designed specifically to support young people in secondary education settings and MHFA England Workplace to support colleagues in workplaces [10]. Despite major policy action, there is currently a lack of evidence-based interventions to improve school staff wellbeing. Therefore, evaluations to establish effectiveness are required.

The Wellbeing in Secondary Education (WISE) intervention is a cluster randomised controlled trial (RCT) that aims to improve teacher and student wellbeing, in addition to teacher performance and attendance at work, and student attendance and attainment. The intervention comprised teacher training in MHFA, a teacher mental health awareness-raising session, and delivery of a staff peer support service [11].

Alongside the main trial, an integrated process evaluation was undertaken to support the interpretation of the main study outcomes and refine the intervention theory [12]. Process evaluations aim to explain how complex interventions work, by examining the processes through which an intervention generated outcomes. This can help explore barriers and facilitators to delivery and uptake that could improve future implementation and scalability [13]. The published protocol for the process evaluation stated that the findings would be reported by the following domains: mechanisms of change and relevant contextual influences; reach; contamination; intervention fidelity; unintended harms; acceptability; and sustainability [12].

The overall aim of the present study was to report process outcomes and measures related to the uptake and fidelity of the MHFA training package, the teacher mental health awareness-raising session, and the staff peer support service within secondary schools in England and Wales. The other parts of the process evaluation—the acceptability of the intervention, and the extent to which the intervention’s mechanisms of change as outlined in the logic model were activated—will be reported in future papers.

We did not use a specific implementation framework or theory for this study. We investigated uptake and fidelity by examining the following aspects of implementation of the WISE intervention, which we assessed through the following objectives:

1. Reach of the WISE intervention training and mental health awareness-raising session
2. Completion of the WISE intervention training (dosage)
3. Fidelity to the planned intervention during delivery of the WISE intervention training
4. Quality of delivery of the MHFA training
5. Fidelity to the planned intervention during the peer support service set-up and ongoing delivery
6. Reach of the peer support service

## Methods

### Sample

Recruitment of state mainstream secondary schools within a 30-mile radius of Bristol, and within the South East and South Central Wales educational consortia was conducted between April and June 2016. These geographical locations were selected due to locality of the research teams, but were broad enough to ensure a wide range of schools in terms of size, socioeconomic status of catchment area and urban versus rural. In Wales, all eligible schools were stratified into three levels according to free school meal eligibility of students (high, medium and low compared to the national average). Two schools were randomly selected from each stratum in each consortium and invited to participate. Schools that declined were replaced by a randomly selected school from the same stratum and region. In England, the study was advertised to head teachers at all eligible schools, and invitations were followed up with relevant senior leaders. Those who expressed interest in participation were stratified into three levels according to free school meal eligibility (high, medium and low compared to the national average) and local authority (Bristol / non Bristol). Where more than two schools fitted into one stratum, two were randomly selected. Once a school was ready to sign up to the study, a research agreement was signed by the team and a head teacher or designated senior leader. This agreement set out the responsibilities of each party and covered data collections, support for the intervention delivery and sharing of findings.

Twenty-five schools were randomly allocated to the intervention or control arm (Supplementary material 1). Further description of the recruitment and randomisation procedures is available [11]. As part of the process evaluation, more detailed data collection was undertaken in four intervention case study schools purposively selected so the sample included a range of geographical areas, student free school meal entitlement and school inspectorate review rating (Ofsted and Estyn for English and Welsh schools, respectively) (Supplementary material 2).

### WISE intervention theory

The WISE intervention is informed by social support theory [14]. Based on findings from the pilot study [15], we hypothesise that peer supporters will provide both emotion-focused and problem-focused support. Perceived availability of social support may be even more important to mental health than actual support [14], and therefore the existence of a peer-delivered support service is theorised to have a positive impact on teacher wellbeing, regardless of actual service utilisation. The programme theory is further informed by an ecological view of school connectedness, which considers the quality of social bonds and interactions within a school to be a characteristic of the whole school environment or culture [14]. Improvement to teachers’ own mental health and wellbeing via supportive relationships with peers should lead to more positive teacher–student relationships [16], which is associated with improved student mental health [17]. Thus, all teachers and students within an intervention school may benefit, regardless of whether they themselves directly engage with the intervention.

### The WISE intervention package

Key features of the WISE intervention:

- Training of 8% of teachers and school staff in Mental Health First Aid
- A mental health awareness-raising session for all teachers
- Delivery of a staff peer support service

A selection of teaching and non-teaching staff at each intervention school were invited to take on the role of peer supporter and, in preparation for this, attended the 2-day standard MHFA training course. To ensure the intervention was delivered with sufficient dose, a minimum 8% of the whole staff body (maximum 16 participants in a group) was required to attend the 2-day standard MHFA training course. Selection of peer supporters was based on nominations by colleagues at the end of the baseline questionnaire; teachers were invited to suggest colleagues that would be well suited to the role of peer supporter. Those with the most nominations were invited to become peer supporters. Where those nominated did not include a range of gender and seniority, other staff with fewer nominations were selected. We did not systematically record each time this happened, but every school involved an element of discussion with the key contact about the list to ensure we had a range of gender, seniority and role (teaching/non-teaching) and every school had at least one change from the original list either due to this discussion, or those approached not wishing to take part.

Following completion of training, attendees were given a short presentation delivered either by the research team or the MHFA trainers, and written guidance on setting up a staff peer support service in their school. The guidance was developed based on pilot study findings [15] regarding factors likely to maximise service usage (see Supplementary material 3).

A shortened version of the youth MHFA course (the 1-day MHFA for schools and colleges) was delivered to a further group of teachers to improve their skills in supporting students in distress. This shortened version was selected as during the pilot it emerged that 2 days training made it difficult for teachers in the target roles such as tutor and year head to attend. Senior leaders were advised to select mainstream teachers who had some pastoral responsibility and who were therefore likely to be in a position to identify students experiencing mental health difficulties (e.g. tutors). A minimum of 8% of all teachers (maximum 16 participants in a group) was required to attend the 1-day MHFA for schools and colleges training course.

Finally, all teachers at the intervention schools were invited to attend a 1-h mental health awareness-raising session, with schools able to choose whether they also made this available to non-teaching staff. This was added to the intervention following pilot findings that many teachers were not aware that the intervention was taking place. There was no target in terms of intervention dose. However, schools were encouraged to schedule the session during staff meetings to ensure the majority of teachers attended. The session covered the importance of teacher and student mental health, tips on how to improve wellbeing and provide initial support to others, information about local help sources and information about the peer support service that was to be established.

All MHFA courses were delivered by MHFA accredited trainers (three in England and six in Wales). Further details of the intervention are available [11]. There was a change to the original protocol which included quantitative data regarding ALGEE use. This was following feedback that it was too difficult to quantify, as peer supporters tended to use some parts of ALGEE, or draw on a general awareness of it, rather than use it or not.

### Data collection methods

A summary of the data collection methods to assess uptake of the intervention is provided (Table 1), and templates of the observational data collection tools and training checklists are provided (Supplementary material 4).

**Table 1:** Data collection methods to assess implementation of the WISE intervention

Research objective	Data source	Informant	Data collection	Timing	Analysis
***Training***					
***1. Reach &*** ***2. Dosage***	Attendance records	Trainers (*n* = 10)	Course registers	During intervention training course	8% of target cohort trained in each school
***3. Fidelity*** ***4. Quality***	Observation of intervention training courses	WISE trainers (*n* = 10), training attendees (2-day standard: *n* = 113; 1-day MHFA for schools and colleges: *n* = 146; intervention case study schools *n* = 4)	Independent assessment of intervention training course by study team (*n* = 2); observation schedules	During intervention training course	Summaries of scores (means, standard deviation, range)
	Fidelity checklist and training materials used	WISE trainers (*n* = 10), training attendees (2-day standard: *n* = 108 (95.6%); 1-day MHFA for schools and colleges: *n* = 118, (80.8%)	Self-assessment; checklists and materials log	During intervention training course	Summaries of scores (means)
	Training evaluation form	Training attendees (2-day standard: *n* = 110 (97.3%); 1-day MHFA for schools and colleges: *n* = 142 (97.3%)	Self-assessment; evaluation forms	Following intervention training	Summaries of scores (means); paired *t*-tests
	WISE trainer interview	WISE trainer (*n* = 6)	Interview	Following intervention training	Thematic analysis
***Peer support service***					
***5. Fidelity***	Peer support feedback and logs	Peer supporters (*n* = 113; intervention schools *n* = 12)	Self-assessment; logs; feedback session hosted by study team	Termly following intervention training course; 2 × feedback sessions	Summaries of responses (counts, percentages)
	Peer supporter and schools MHFA attendee focus groups	Training course attendees (*n* = 4–8 staff, intervention case study schools *n* = 4)	Focus group	6 months post-training; 18 months post-training	Thematic analysis
***6. Reach***	Teacher questionnaires	Teachers at intervention schools (12-month follow-up *n =* 557, 24-month follow-up (*n* = 510)	Survey questions regarding use of peer support service	12-month follow-up (teachers); 24-month follow-up	Summaries of scores (counts, percentages)

Research objectives: 1. Reach of the WISE intervention training, mental health awareness-raising session and the peer support service; 2. Completion of the WISE intervention training (dosage); 3. Fidelity to the planned intervention during delivery of the WISE intervention training; 4. Quality of delivery of the MHFA training; 5. Fidelity to the planned intervention during the peer support service set up and ongoing delivery; 6. Reach of the peer support service

#### Research objectives 1 and 2: Reach and dose of the training

To measure the reach and dosage of the MHFA training, the numbers of teachers and other school staff attending and completing the MHFA training courses were recorded. Reach of the 1-h awareness-raising course was measured by recording the number of staff that attended each one; however, it was not possible to know how many of these were teachers and how many were other staff.

#### Research objectives 3 and 4: Fidelity and quality of the training

Fidelity to the training delivery plan and quality of the training was measured in three different ways:

1. Observations of the training

Observations of the 1-h mental health awareness-raising session, the 2-day standard MHFA and the 1-day MHFA for schools and colleges training course were undertaken in the four intervention case study schools. Two members of the research team independently observed all sessions; data related to the second observation in one case study school was misplaced by a member of the study team. Standardised observation schedules were completed to assess coverage of materials, quality of delivery and participant engagement for each section of the training. Scales were constructed by the study team based on discussion with MHFA England as to what the key content of the training is and how the trainers themselves are assessed when becoming accredited. Items were measured using a 5-point Likert scale (1 = very poor; 2 = poor; 3 = neither poor nor good; 4 = good; 5 = very good). Coverage of key topics was assessed through a binary measure (“yes” or “no”) (Supplementary material 4).

2. Training checklists and evaluation forms

Following completion of both MHFA training courses, attendees at the 12 intervention schools were asked to complete a study-specific checklist devised by the research team, recording the content delivery (each item assessed as covered or not) and quality of the training in terms of trainer knowledge and skills, and modes of learning utilised (e.g. group work, presentation of slides). Each quality measure was assessed using a 5-point Likert scale (1 = very poor; 2 = poor; 3 = neither poor nor good; 4 = good; 5 = very good).

Attendees of all MHFA training in the 12 intervention schools completed standardised MHFA evaluation forms (Supplementary material 4). These forms recorded self-assessed knowledge and confidence to support others before and after the course, and views on course quality (5-point Likert scale) (1 = very poor; 2 = poor; 3 = neither poor nor good; 4 = good; 5 = very good) (Supplementary material 4).

3. Trainer interviews

Semi-structured interviews (*n* = 6) were conducted with a subgroup of the trainers. Trainers were purposively sampled to ensure that a representative from each of the 12 intervention schools was interviewed. These interviews enabled triangulation of the quantitative and qualitative data collected from training attendees, by providing a different perspective, and also enabling comparison of these sessions to others that the trainers had delivered previously. They explored experiences of delivery, fidelity and motivations for any adaptations undertaken.

#### Research objective 5: Fidelity of the peer support service

In all intervention schools, a convenience sample of 1–2 peer supporters were invited to attend a feedback meeting with the study team approximately 6 and again 18 months after training. A member of the research team went through a structured list of questions that assessed adherence to each item of the peer support service guidance. Additionally, in case study schools, a convenience sample (based on their availability to attend) of peer supporters took part in focus groups (*n* = 8) held at their school. These took part approximately 6 and 12 months post-intervention delivery. The focus groups explored their experiences, and the associated barriers and facilitators to implementation of the peer support service.

#### Research objective 6: Reach of the peer support service

We were unable to recruit peer support users to take part in an interview. This research objective was therefore assessed in the following two ways:

1. Teacher questionnaires

All teachers in intervention schools were asked to complete anonymised questionnaires regarding their use of the peer support service at the 12- and 24-month follow-up time-points.

2. Peer supporter logs

Peer supporters were also asked to complete an electronic log three times a year documenting delivery of support to colleagues in the previous 2 weeks. To mitigate against the risk of seasonal bias (e.g. stress associated with end of term examinations), peer support logs were issued at different times during the academic term. The log assessed reach (number of staff supported), the broad demographic and professional characteristics of the staff members supported, type of problem addressed (e.g. work or personal) and outcome of the interaction. The log was also used to record when peer supporters had left the school.

#### Analysis

Qualitative and quantitative data were analysed separately and then the findings were triangulated across all relevant datasets for each research objective to enable a full interpretation incorporating all participant perspectives.

MHFA evaluation forms, checklists, teacher questionnaires and observation schedules were analysed descriptively using counts, percentages, means, standard deviations and ranges (Table 1). Ordinal data from 5-point Likert scales for the observational schedules were presented as means scores by observer and across all sections of the course and percentage of responses categorised as “good” or “very good”. Quantitative analyses were undertaken using the STATA statistical package, release 14 (STATA Corp, College Station, TX).

Interviews with trainers and peer supporters were audio recorded and transcribed verbatim. Any potentially identifying information was removed. Interview data were analysed using thematic analysis [18]. Separate coding trees were developed through an iterative process for each dataset. Independent coding of two transcripts for each dataset was undertaken. A priori codes that map onto the process evaluation domains were included in the initial coding trees, along with novel codes that emerged from the data. As subsequent codes were generated from the rest of the transcripts, the coding trees were adapted to include them. Codes and their meaning were agreed between team members in an ongoing dialogue, while allowing sufficient interpretive flexibility to enable generation of themes. Codes were then assembled into themes; candidate themes were reviewed, refined and confirmed by the team, and then compared across datasets. Qualitative analyses were assisted with the QSR NVivo11 software.

To examine implementation at the school level, we classified all schools according to reach, dose and fidelity. Schools covered a range of adherence to these features of implementation, rather than clearly falling into high versus low implementation groups (Supplementary material 5). There was no evidence of systematic variation in implementation, or patterns in school characteristics that would explain this.

## Results

### Mental Health First Aid training findings

#### Research objectives 1 and 2: Reach and dosage

Across the 12 schools assigned to the intervention arm, 113 (8.6%) teachers and support staff attended and completed the 2-day standard MHFA training, and 146 (11.1%) teachers and support staff completed the 1-day MHFA for schools and colleges. Six hundred and sixty-six (54.5%) teachers and school staff attended the 1-h awareness-raising session. In eight (66.7%) of the 12 intervention schools, the pre-specified intervention dose (at least 8% of school staff attending the course and becoming a peer supporter) of 2-day standard MHFA training was achieved. One additional staff member was required to be trained in each of the remaining four schools to reach sufficient dose. In nine (75.0%) of the intervention schools, the pre-specified 8% of teachers attended the 1-day MHFA for schools and colleges training course. Of the three schools not reaching sufficient dose, an average of two additional teachers would have been needed to be trained to achieve the required dose. Reasons for schools not achieving sufficient dose included researcher error in calculating the numbers, nominated staff unable to attend at the last minute and trained staff leaving the school shortly after training.

#### Research objectives 3 and 4: Fidelity and quality of training

1. Observer assessed

In the four case study schools, observer-assessed fidelity and quality of delivery of training was consistently high for each of the items of assessment (instructor knowledge of materials, presentation skills, facilitation and support of the learning, interest from the group and coverage of content). There was little variation in mean scores on these items for the 1-day MHFA for schools and colleges (range 3.9–4.3 out of 5) and 2-day standard MHFA training course (range 4.1–4.4), although mean scores were slightly lower for the 1-h mental health awareness-raising session (range 3.4–4.1) (Table 2).

2. Participant assessed

**Table 2:** Observer-rated fidelity and quality of delivery of the MHFA training package at case study schools (mean scores by observer and across all sections of the course)

	One-day MHFA for schools and colleges			Two-day standard MHFA training			Raising awareness session		
	***N*** = 7			***N*** = 8			***N*** = 7		
	Mean (SD)	Range	Percent*	Mean (SD)	Range	Percent*	Mean (SD)	Range	Percent*
*Observer fidelity checklist forms*									
Knowledge of materials	4.2 (0.9)	2.8–5.0	78.9	4.4 (0.7)	3.0–4.9	85.1	4.1 (0.7)	3.3–5.0	77.4
Presentation skills	4.3 (0.6)	3.5–5.0	94.3	4.1 (0.6)	4.0–5.0	80.3	3.8 (0.7)	2.8–4.9	65.5
Facilitation and support of the learning	4.0 (0.8)	2.5–5.0	88.6	4.1 (0.7)	3.0–4.9	78.4	3.6 (0.6)	3.1–5.0	54.8
Interest from the group	3.9 (0.3)	3.5–4.3	84.0	4.3 (0.5)	3.7–5.0	84.3	3.4 (0.4)	3.0–4.1	38.1
Coverage of material (yes)			94.0		84.2–100.0%	96.3			100.0

*SD* standard deviation. Items measured using a 5-point Likert scale. *Percentage of responses rated as “good” or “very good”

One hundred and eighteen of the 146 (80.8%) attendees of the 1-day MHFA for schools and colleges and 108 of 113 (95.6%) attendees of the 2-day standard MHFA training course completed a participant checklist. Most attendees scored trainers highly for knowledge of materials, presentation skills, diversity of learning materials, communication skills, use of a range of teaching approaches and ability to keep the course focused and relevant. There was little variation in the mean scores for each of the instructor qualities by the 1-day MHFA for schools and colleges (range 4.6–4.7) and 2-day standard MHFA (range 4.6–4.7) training courses (Table 3).

**Table 3:** Participant-assessed quality of training and fidelity of the MHFA training package

	One-day MHFA for schools and colleges			Two-day standard MHFA training		
	Mean (SD)		Percentage*		Mean (SD)	Percentage*
*MHFA training evaluation forms*	***n***	***N*** **= 146**		***n***	***N*** **= 113**	
Overall	138	4.6 (0.5)	97.9	110	4.5 (0.6)	97.3
Presentation slides	140	4.4 (0.6)	94.4	109	4.3 (0.6)	90.0
Video clips	140	4.7 (0.5)	99.3	110	4.5 (0.6)	97.3
Manual	123	4.7 (0.5)	99.3	110	4.7 (0.5)	100.0
Learning exercises	136	4.4 (0.6)	96.5	110	4.5 (0.6)	96.4
Environment	138	4.1 (0.7)	88.0	107	3.8 (0.9)	73.6
Structure	140	4.4 (0.6)	95.1	109	4.4 (0.7)	95.5
Content	140	4.6 (0.6)	97.2	109	4.6 (0.6)	97.3
*Participant fidelity checklist forms*	***n***	***N*** **= 118**		***n***	***N*** **= 108**	
Knowledge of materials	106	4.6 (0.7)	94.1	103	4.7 (0.5)	98.2
Presentation skills	107	4.7 (0.6)	97.5	103	4.7 (0.6)	95.4
Diversity of learning materials	107	4.6 (0.7)	96.6	103	4.6 (0.6)	96.3
Communication and interaction	107	4.7 (0.6)	98.3	103	4.7 (0.6)	99.1
Facilitation and support of the learning	107	4.7 (0.6)	98.3	102	4.6 (0.6)	100.0
Relevance of content and discussion	107	4.7 (0.6)	96.6	103	4.7 (0.5)	100.0
Flexibility of use of most relevant materials	107	4.7 (0.6)	97.5	103	4.7 (0.6)	100.0

*SD* standard deviation. Items measured using a 5-point Likert scale. *Percentage of responses rated as “good” or “very good”

One hundred and forty-two of the 146 (97.3%) attendees of the 1-day MHFA for schools and colleges and 110 of 113 (97.3%) attendees of 2-day standard MHFA training course completed a MHFA training evaluation form. In keeping with observer-assessed quality of training, overall mean scores for the 1-day MHFA for schools and colleges training courses were high (range 4.4–4.7), with the exception of slightly lower scores for participant rating of environment in which the training was delivered (4.1, SD: 0.7). Mean scores for the 2-day standard MHFA course were also high (range 4.3–4.7), with lower scores observed for participant rating of training course environment (3.8, SD 0.9) (Table 3).

3. Trainer assessed

Despite different levels of experience in delivering MHFA sessions, all six trainers reported high levels of fidelity in terms of ensuring key content was delivered. However, three main factors appeared to present a challenge to fidelity, requiring trainers to deliver the course with flexibility, while still ensuring all key content was covered. These factors were needs of the group, location of the training and scheduling within the school day.

*Needs of the group*

Trainers discussed the need to exhibit flexibility in relation to choice of materials or timetabling of exercises depending on the needs of the group: “You’re not meant to go off the planned route really but if the room is slumping slightly you can kind of get them sort of re-energised for a little while and get them involved in something” [Trainer five]. They also used their skill as trainers to note and respond to dynamics within the group, to help ensure more effective participation by attendees: “I think it’s a general thing about watching your group, seeing how they’re interacting, and making sure that they are interacting about the subject matter” [Trainer three].

*Location of the Mental Health First Aid training delivery*

Delivery of the MHFA training package usually took place on the school site, either during an in-service training (INSET) day (for the 1-day training course) or usual school day. However, being on-site resulted in interruptions to the delivery of training in some schools, due to competing priorities of school staff, such as resolving student incidents, performance management meetings and break duties: “There was an incident in the school that afternoon, which required several members of staff to have to leave in the afternoon and go and do things and come back. I guess that’s just the nature of life inside a school” [Trainer two]. In such situations, trainers again discussed being flexible in delivery during such interruptions, to ensure coverage of sufficient content: “Frequently I was having to move the day around or rejig, to make sure they covered the most important points” [Trainer three].

*Scheduling MHFA training within the school timetable*

The school timetable presented challenges to fidelity of the MHFA training package. Often trainers reported a reduction in time available due to expectations of delivering the course within a school day, with set break and lunchtimes and other scheduled school events being prioritised: “We couldn’t start at eight thirty because it was an inset day and the Principal wanted staff to come and join the main assembly for a talk. So that pushed it beyond nine o’clock” [Trainer four]. This required trainers to be adaptive in their delivery style to ensure that key materials were covered within a shorter timescale: “We’re not going to be pedantic about timescales…we’ll just go with the flow of the school day and just stop and start when it automatically fits” [Trainer six].

The trainers reflected on the 1-h awareness-raising session and perceived that it was PowerPoint heavy and not necessarily conducted at an optimum time (e.g. at the end of the school day) for the school staff to remember and fully process the messages within the session. However, one particular trainer used personal experience to engage the school staff and make the messages less abstract. It was also felt that there was not enough time built in for it to be interactive and allow discussions: “They were struggling a little bit at the end of the day. There was a lot of PowerPoints and quite a lot of actually reading from the PowerPoints. The main bits that actually worked were when I handed out the little sticky post-it notes to get them to do things... I used my own experience of bipolar disorder to cover the stigma section, which completely changed the dynamic in the room” [Trainer one].

The trainers’ reports of high fidelity were corroborated by observers’ feedback forms which confirmed that the core content was covered. The trainers’ descriptions of the skill they used to be flexible and response to the needs of the group and practical considerations of the school context were corroborated by both observers and attendees high rating of trainers’ skills and quality of the sessions.

#### Research objective 5: Fidelity to guidance (peer support service)

At the 6-month peer supporter feedback meetings, nine (75.0%) of the intervention schools indicated that support had already been provided to colleagues by peer supporters. Peer supporters at the majority (*n* = 9, 75.0%) of schools had met as a group to discuss the set-up of the staff peer service, with some schools indicating they had held regular up-date meetings since then (*n* = 5, 41.7%). Usually peer supporters provided support to each other through an informal “buddy” system (*n* = 11, 91.7%), although one school (8.3%) reported implementing a more formal approach. Five (41.7%) of the schools had set-up a formal confidentiality policy for the peer support service at this point.

All schools used advertising to launch the peer support service. Methods to promote the service included posters provided by the research team (*n* = 10, 83.3%), newsletters (*n* = 1, 8.3%), staff briefings (*n* = 11, 91.7%), staff email (*n* = 4, 33.3%) and posting information in staff pigeon holes (*n* = 1, 8.3%). Half of the schools planned to advertise the service at the start of the following academic year (8 to 11 months after delivery of MHFA training). All schools (*n* = 12, 100.0%) offered service users the choice of which peer supporter they contacted. Most schools offered a confidential space where support could be provided (*n* = 9, 75.0%) and senior leaders had helped to raise the profile of the peer support service (*n* = 8, 66.7%).

The second feedback meeting took place at only ten (83.3%) schools (approximately 18 months after training). Since the first feedback meeting, none (0.0%) of the schools had met as a group, with most (*n* = 9, 75.0%) mentioning just discussing any issues with other peer supporters informally if needed. Three (25.0%) of the schools had re-advertised the service, by email (25.0%), posters (1, 8.3%) and through a staff newsletter (1, 8.3%). Peer supporters in fewer schools indicated that they perceived they had senior leadership support at the second feedback meeting (4, 33.3%). During feedback meetings, peer supporters indicated varied levels of involvement from the senior leadership team. Although the majority indicated that additional support from senior leadership would be advantageous in terms of keeping wellbeing on their agenda, some peer supporters perceived their involvement would be inappropriate.

Qualitative data from trainer interviews and peer supporter focus groups shed light on the challenges in setting up the peer support service. In some cases, a short amount of time was found at the end of the second training day for the group to begin to discuss the service, but this was limited: “…it might have prompted a little bit more conversation and discussion about what do we do? But there wasn’t a huge amount of that and the course doesn’t really lend itself, because again, you’ve got to get through this and that” [Trainer four].

Difficulty in finding further time to meet was noted as the reason that some groups failed to meet at all even to set the service up, and no groups were meeting a year on:

Peer Supporter: “If we’ve got half an hour free at all it will be different times in the day.”

Interviewer: “Have you met as a whole group or is it difficult with the time?”

Peer Supporter: “No, not as a whole group. We had a few meetings in the term after the training, but even then it was a real struggle to get people. And once you get the same people over and over, you start to think, well it’s not good” [School 1D, phase two].

This may have impacted the uptake of the intervention as the peer supporters did not have the space to reflect on their practice and the service and discuss any improvements that could be made.

The guidance (Supplementary material 3) was deliberately flexible, to ensure the peer support service could be implemented in a realistic and sustainable way in each school context. But one trainer observed that in at least one group this added an additional complexity to the peer support role that may have been counter-productive to getting the service going: “…they got really bogged down in policy and procedure and then some people said, well I’m not going to be comfortable doing this if, I want to know” [Trainer six].

It was reported by some peer supporters that there was a struggle to find the time and space to meet with staff who wanted support. Some reflected that it is hard to find a confidential space within a school as many of the spaces have staff and students coming and going on a regular basis. This could have had an effect on the staff approaching peer supporters and the quality of the conversation undertaken: “And also, finding a place at that time as well… I was seeing someone after school, and we were chatting, talking about something they were a bit concerned about, and then somebody else just walked in and just stood there. I didn’t want to say, this is a private, a mentoring, this is confidential. So this person doesn’t want me telling somebody else that, so that was difficult……I didn’t know what to do because I didn’t want to embarrass the person that was there, I wanted to be rude to the person who just stood there but I couldn’t, and they still didn’t go, they still didn’t get the message” [School 2 L, phase two].

Although most services were delivered on an ad hoc basis, as and when colleagues approached a supporter for help, in one of the case study schools the peer supporters also created a specific space and time that the staff knew they were available to access should they need support, which may have avoided the above problem. However, they found this difficult to implement due to the additional demands it placed on their time: “I mean when we first did it, there was talk about having like a drop in, and then we were going to kind of have a rota and do that. But, you know, people are just so busy that it’s hard to ask people to give up their free time” [School 1D, phase two].

A number of comments suggested that to address some of these implementation problems such as lack of time and lack of clarity over policies, stronger support and recognition from senior leadership was needed: “And I think that maybe needs to be addressed because we want to have more of an impact. Then actually, we need to have that recognition, as to the role that we are playing. And perhaps sitting down with the Head and, as a group of people, this is our plan, how will you support us, kind of thing because it is really important” [School 2 L, phase 1].

#### Research objective 6: Reach of peer support service delivery

At the 12-month teacher questionnaire follow-up, 34 (6.1%) of 557 teachers indicated they had accessed the peer support service in the previous 12 months. Most frequently, teachers indicated they had used the service once or twice in the academic year (*n* = 16, 47.1%) or once a term (three times a year) (*n* = 9, 26.5%). Similarly, at the 24-month teacher questionnaire follow-up, only a small proportion (*n* = 30, 5.9%) of 510 teachers indicated they had accessed the peer support service in the previous 12 months (Table 4).

**Table 4:** Teacher-reported use of the staff peer support service at follow-up time-points

	12-month follow-up	24-month follow-up
	***N*** = 557	***N*** = 510
	***n*** (%)	***n*** (%)
*Use of staff peer support in previous year?*		
Yes	34 (6.1)	30 (5.9)
No	523 (93.9)	480 (94.1)
*Frequency of use*		
Once or twice	16 (47.1)	13 (43.3)
Once a term	9 (26.5)	6 (20.0)
Once or twice a month	5 (14.7)	8 (26.7)
More than once a week	4 (11.7)	3 (10.0)
*Usefulness of service*		
Not at all helpful	1 (2.9)	2 (6.9)
It helped	18 (54.6)	14 (48.3)
It helped a lot	14 (42.4)	13 (43.3)

Of the 113 peer supporters trained in the intervention schools, over half (*n* = 60.6, 53.6%) completed logs at each of the five time-points, and each supporter completed a mean of 3.1 (SD 1.5) logs. Sixteen (*n* = 14.5%) peer supporters did not complete a log at any time-point. Ninety-two (81.4%) peer supporters were still employed by the schools at the final data collection time-point. The mean number of logs completed by school varied (range 2.2 to 10.6) and decreased slightly over time (mean difference between first and last data collection 0.7).

Across all time-points combined, peer supporters reported that they had supported a mean of 1.7 (SD 1.8) colleagues in the previous 2 weeks, of which approximately half (mean 0.7, SD 0.8) were additional colleagues who they would not have supported prior to being trained. Most often, support was provided to each person once (mean number of colleagues helped once at each time-point (23.8, 40.8%) or twice (18.8, 32.2%)). The peer supporters reflected that they were unsure about which contacts should be recorded as part of the intervention, and which would have happened anyway outside of their peer support role. This meant that the logs may inaccurately estimate the work of the peer supporters: “The struggle for me is how do you know if they’re coming to you as a peer mentor or, how do you know if they’re coming to you because they would come to you anyway. Measuring that, you know. Quantifying Peer Supporter support versus pretty much we were all doing that anyway” [School 4N, phase 1].

## Discussion

The findings from this process evaluation examining uptake and fidelity of the WISE intervention, designed to improve the emotional wellbeing of secondary school teachers and students, indicate that it was delivered with reasonably high fidelity, and a staff peer support service was established with general, but not complete, adherence to guidelines. In some schools insufficient numbers of staff received MHFA training and extent of delivery of the peer support service compromised intervention dose and reach. Although there are no published studies of teacher peer support services, a previous randomised controlled trial found an effect when 15% of the student body was trained to be a peer educator [19]. We were unable to meet this threshold in relation to training of teachers in the study schools for this study. The findings presented here that participants were positive about the content and delivery of the MHFA training resonates with other studies [20, 21]. However, there were challenges in terms of delivery in the school context with the risk of interruptions and timetabling issues. While this did not appear to impact on fidelity or quality due to the flexibility of the trainers, it may have meant that some participants were distracted or missed parts of the training. Holding training off-site may give teachers time to learn without interruption; however, schools may not always be able to re-arrange duties to accommodate.

Implementation of the peer support service varied between schools. A service was set up in all twelve intervention schools, and there was evidence that all were made use of by some staff. Support was most commonly provided to teachers, face-to-face, and on an ad hoc basis. However, reach of the peer support according to teachers was low and peer supporter drop-out created further dosage reductions in some schools. Further, there appeared to be a loss of momentum in relation to delivery, with few schools reporting that they re-advertised the service in the subsequent academic year and support from senior leaders, by visibly championing the service and supporting its delivery in practical terms, appeared to dwindle. Both these features of the service were recommendations to encourage longer-term sustainability of the peer support service, identified as part of the qualitative evaluation comprising the pilot study [15]. However, implementation of these recommendations does not appear to have been fully realised in the present study. Any intervention targeting teacher or student mental health needs to have genuine support from senior leadership, for example ensuring it has a raised profile and allowing staff adequate time to engage with it.

These issues may have contributed to barriers to service use highlighted by trainers and peer supporters, namely concerns about confidentiality, concerns that peer supporters did not have time or a safe space to provide support, and not knowing about the service. As a result, although all teachers did have increased access to support, they may not have always considered this an acceptable form of support, which contributed to low service use. The importance of having an intervention champion and support from senior management has been identified as key for success in the child and adolescent health literature [22]. Whether these barriers to uptake of the intervention had an impact on effectiveness, either of the whole intervention or of the different components, will be reported in a future outcomes paper.

### Comparison with the literature

The findings presented here that participants were positive about the content and delivery of the MHFA training resonates with other studies [20, 21]. Taken together, these results indicate that it is feasible to deliver high quality and consistent MHFA and mental health awareness training to groups of staff within a school setting, although maximising numbers can be challenging. This is relevant to current UK policy in which there is an increasing emphasis for schools to play a key role in supporting children and young people’s mental health [23].

The peer support service was not implemented with the same consistently high fidelity and was not highly used. Other evaluations of peer support work have uncovered similar challenges in terms of implementation and identify the need for such services to have clear expectations around the role and how it fits within the wider organisation [24, 25]. This did not appear to have been achieved well in this study, as peer supporters discussed the difficulties of meeting together, and identifying time and space to deliver the service. Given the high workloads and time pressured roles of schoolteachers, is it likely that such a service could only ever be more fully embedded into the organisation and acknowledged as part of individual workers’ roles with greater prioritisation from senior leaders than was evident here. Previous studies have reported that school level factors, which may include how far senior leaders are concerned about mental health, can interfere with the successful uptake of interventions aiming to support the mental health or staff or students [26–28]. Further research to understand how schools and senior leaders view teacher mental health in relation to the broader organisational priorities, and how to encourage a high level of buy-in for interventions to support teachers, is needed. This will help intervention developers ensure that such interventions are acceptable to senior leaders and are feasible not only to initially implement, but to fully embed and sustain within the wider system.

Complex interventions may often be tailored when being implemented in different contexts. Process evaluations can usefully identify whether delivery occurred as intended, and the impact on desired (and undesired) outcomes [29]. For example, a study relating to a trial of a school-based prevention programme delivered in the USA showed differential intervention effects in different schools. The authors suggested this was related to higher quality of implementation and senior leader support [30]. The findings reported here indicate a high level of fidelity, but with intervention dose and reach lower than anticipated. However, as part of our analysis of the main trial and economic analyses, we will undertake exploratory analyses to assess the impact of differing levels of implementation of the mental health training package and peer support service on corresponding improvements to health outcomes.

### Strengths and limitations of the study

To our knowledge, this is the first study that reports the uptake and fidelity of a mental health training package and staff peer support service aiming to improve the wellbeing of secondary school teachers. We collected extensive process data using qualitative and quantitative data collection techniques related to delivery of the MHFA and 1-h mental health awareness training and implementation of the staff peer support service, to provide evidence as to the extent that the WISE intervention was delivered as intended.

There are some limitations to the study. As the process data were collected from intervention schools only, it was not possible to blind researchers during data collection. However, analysis was undertaken to address research questions that were established a priori, mitigating the risk of reporting bias. As study researchers undertook interviews and focus groups, participants may have been influenced to respond more positively. Researcher observations of delivery of the MHFA training package occurred in case study schools only. Therefore, observer-rated quality and fidelity reported in the present study may not be representative of the experience of the other intervention schools participating in the study. However, fidelity checklists completed by course attendees indicated training quality to be similarly high across intervention schools. In addition, comments from feedback meetings and MHFA training forms were largely similar between the case study and other intervention schools suggesting that the case study schools were not systematically different from the rest of the intervention schools.

Of the 113 participants trained as peer supporters, a substantial proportion did not complete a log at any time-point (16, 14.5%) or only completed a log at one time-point (20, 17.7%). Disengagement with completion may be unrelated to delivery of the peer support service and may relate to the confusion about precisely what kind of contact should be recorded. We cannot ascertain whether or not these individuals had a less active role as peer supporters, and whether the peer support service was delivered by fewer than intended individuals.

There was also discordance between teacher and peer supporter-reported use of the service making it difficult to accurately report the reach of the intervention. Due to the informal nature of delivery, teachers may not have been aware that they were using the peer support service or may not be willing to disclose that they had. Peer supporters could over-inflate reported use, although the recorded confusion about which contacts warranted recording suggests, if anything, that use of the service was probably under-recorded. We were unable to recruit users of the peer supporters to take part in the study. Therefore, their views in relation to how helpful the peer support service was and whether the peer supporters appeared to make use of the MHFA training are not captured in the study.

## Conclusion

The findings suggest that it is possible to deliver mental health training such as MHFA with fidelity and high quality to groups of staff within secondary schools. However, in this study, fewer staff than intended received MHFA training in a minority of schools, and the peer support service activity in terms of regular meetings and advertising waned through the 2-year study period. Future studies should explore how to better ensure visible and ongoing support from senior leadership for any mental health intervention within schools, in order to maximise successful intervention uptake. School leaders wishing to introduce new mental health initiatives need to ensure that they are prioritised and that sufficient resource is made available, including dedicated staff time, if they are to be successfully implemented.

Further, the low reach of the peer support service according to teachers’ self-report highlights the need for further work to explore other ways to support teachers’ mental health that might have greater reach. The reasons for this apparently low uptake, and the extent to which the intervention had an impact on teacher and student wellbeing and mental health, will be reported in future papers.

## Supplementary information

**Additional file 1.** Baseline school variables (for academic year just completed – 2015-2016, pre intervention). Table of characteristics.

**Additional file 2.** Sample for case study schools. Characteristics of case study schools.

**Additional file 3.** Guidelines for peer supporters. Guidelines for peer supporters to set up a peer support service in the school as part of the WISE intervention.

**Additional file 4.** Data collection tools. Data collection tools: observation schedule template, mental health first aid evaluation forms, participant assessed fidelity check lists.

**Additional file 5.** Extent of implementation of the WISE intervention at school-level. Table indicating whether domains of fidelity, reach and dosage were achieved by intervention schools.

## Data Availability

The datasets used and/or analysed during the current study are available from the corresponding author on reasonable request.

## Abbreviations

MHFA: Mental Health First Aid
WISE: Wellbeing In Secondary Education
RCT: Randomised controlled trial
SD: Standard deviation
INSET: In-service training

## References

[1] Stansfeld S (2011). Occupation and mental health in a national UK survey. Soc Psychiatry Psychiatr Epidemiol.

[2] Kidger J (2016). Teachers’ wellbeing and depressive symptoms, and associated risk factors: A large cross sectional study in English secondary schools. J Affect Disord.

[3] Melchior M (2007). Work stress precipitates depression and anxiety in young, working women and men. Psychol Med.

[4] Harding S (2019). Is teachers’ mental health and wellbeing associated with students’ mental health and wellbeing?. J Affect Disord.

[5] Kidger J, et al. The effect of the school environment on the emotional health of adolescents: a systematic review. Pediatrics. 2012; 10.1542/peds.2011-2248. 10.1542/peds.2011-2248 22473374

[6] Kelly C, Jorm A, Wright A (2007). Improving mental health literacy as a strategy to facilitate early intervention for mental disorders. Med J Australia.

[7] May T (2017). The shared society: Prime Minister’s speech at the Charity Commission annual meeting.

[8] Kitchener B, Jorm A (2002). Mental health first aid training for the public: evaluation of effects on knowledge, attitudes and helping behavior. BMC Psychiatry.

[9] Jorm A (2010). Mental health first aid training for high school teachers: a cluster randomized trial. BMC Psychiatry.

[10] Forbes T (2015). Vaccination uptake by vaccine-hesitant parents attending a specialist immunization clinic in Australia. Hum Vaccin Immunother.

[11] Kidger J (2016). Protocol for a cluster randomised controlled trial of an intervention to improve the mental health support and training available to secondary school teachers – the WISE (Wellbeing in Secondary Education) study. BMC Public Health.

[12] Evans R (2018). A cluster randomised controlled trial of the Wellbeing in Secondary Education (WISE) Project–an intervention to improve the mental health support and training available to secondary school teachers: protocol for an integrated process evaluation. Trials.

[13] Moore G (2014). Process evaluation in complex public health intervention studies: the need for guidance. J Epidemiol Community Health.

[14] Thoits P (2011). Mechanisms linking social ties and support to physical and mental health. J Health Soc Behav.

[15] Kidger J (2016). A pilot cluster randomised controlled trial of a support and training intervention to improve the mental health of secondary school teachers and students – the WISE (Wellbeing in Secondary Education) study. BMC Public Health.

[16] Jennings P, Greenberg M (2009). The prosocial classroom: teacher social and emotional competence in relation to student and classroom outcomes. Rev Educ Res.

[17] Marlow R (2013). Influence of problematic child-teacher relationships on future psychiatric disorder: population survey with 3-year follow-up. Br J Psychiatry.

[18] Braun V, Clarke V (2006). Using thematic analysis in psychology. Qual Res Psychol.

[19] Campbell R (2008). An informal school-based peer-led intervention for smoking prevention in adolescence (ASSIST): a cluster randomised trial. Lancet.

[20] Borrill J (2010). Mental Health First Aid Training: Initial Evaluation by Private Sector Participants.

[21] Brandling J, McKenna S (2010). Evaluating Mental Health First Aid training for line managers working in the public sector.

[22] Durlak J, DuPre E (2008). Implementation matters: a review of research on the influence of implementation on program outcomes and the factors affecting implementation. Am J Community Psychol.

[23] Department of Health, Transforming Children and Young People’s Mental Health Provision: a Green Paper, 2017. Available from: https://www.gov.uk/government/consultations/transforming-children-and-young-peoples-mental-health-provision-a-green-paper. Accessed 24 Aug 2020. 10.1177/1367493518786021 30041539

[24] Moran G (2013). Challenges experienced by paid peer providers in mental health recovery: a qualitative study. Community Ment Health J.

[25] Mancini M (2018). An exploration of factors that effect the implementation of peer support services in community mental health settings. Community Ment Health J.

[26] Allen K, et al. Teachers’ perceptions of the impact of the Incredible Years® Teacher Classroom Management programme on their practice and on the social and emotional development of their pupils. Br J Educ Psychol. 2019;90(S1):75–90. 10.1111/bjep.12306 31297801

[27] Wilde S (2019). Mindfulness training in UK secondary schools: a multiple case study approach to identification of cornerstones of implementation. Mindfulness.

[28] Ouellette R (2018). Teacher job stress and satisfaction in urban schools: Disentangling individual-, classroom-, and organizational-level influences. Behav Ther.

[29] Moore G (2015). Process evaluation of complex interventions: Medical Research Council guidance. BMJ.

[30] Kam C-M, Greenberg MT, Walls C (2003). Examining the role of implementation quality in school-based prevention using the PATHS Curriculum. Prev Sci.

