# Supplementary material for: Delivery of a Mental Health First Aid training package and staff peer support service in secondary schools: a process evaluation of uptake and fidelity of the WISE intervention
Source: Trials. 2020 Aug 26;21:745. doi: 10.1186/s13063-020-04682-8 (PMC7448323; doi:10.1186/s13063-020-04682-8)
Supplement: Supplementary file 1 — Additional file 1. Baseline school variables (for academic year just completed – 2015-2016, pre intervention). Table of characteristics. [file 13063_2020_4682_MOESM1_ESM.docx]

Supplementary material 1. Baseline school variables (for academic year just completed – 2015-2016, pre intervention)

|  | **Control** | | | **Intervention** | | |
| --- | --- | --- | --- | --- | --- | --- |
| School level variables: | **N** | **Mean/ Median / %** | **SD / IQR / range** | **N** | **Mean /Median / %** | **SD / IQR / range** |
| Median teacher student ratio | 13 | 0.33 | (0.31-0.36) | 12 | 0.35 | (0.31-0.39) |
| Median % of teachers retired | 12 | 1.7 | (0.7-3.2) | 9 | 0 | (0.0-1.8) |
| Median % of teachers left for other reasons | 12 | 14.6 | (7.3-21.4) | 9 | 21.6 | (11.7-25.0) |
| Mean number of teachers in whole school | 13 | 60.1 | Range: 33-95 | 12 | 60.0 | Range: 27-101 |
| FSM tertile (%)   - Low - Middle - High | 13 | 3 (23%)  6 (46%)  4 (31%) |  | 12 | 6 (50%)  2 (17%)  4 (33%) |  |
| Mean number of students in whole school | 13 | 834.3 | Range: 484-1,203 | 12 | 907.2 | Range: 389-1584 |
| Median % teacher absence | 12 | 6.1 | (3.7-12.8) | 8 | 4.0 | (3.5-10.1) |
| Median % student attendance | 13 | 93.6 | (93.4-94.8) | 12 | 93.7 | (93.2-94.8) |
| Student attainment (>= average) (%) | 13 | 4 (30%) |  | 12 | 5 (42%) |  |
