# Supplementary material for: Delivery of a Mental Health First Aid training package and staff peer support service in secondary schools: a process evaluation of uptake and fidelity of the WISE intervention
Source: Trials. 2020 Aug 26;21:745. doi: 10.1186/s13063-020-04682-8 (PMC7448323; doi:10.1186/s13063-020-04682-8)
Supplement: Supplementary file 2 — Additional file 2. Sample for case study schools. Characteristics of case study schools. [file 13063_2020_4682_MOESM2_ESM.docx]

Supplementary material 2. Sample for case study schools

| **School** | **Trial Status** | **Site** | **Administrative Region** | **FSM Eligibility** | **School Size** | **Inspectorate Assessment** |
| --- | --- | --- | --- | --- | --- | --- |
| School 1 | Intervention | England | 1 | Low | Large | Good |
| School 2 | Intervention | England | 2 | High | Small | Requires improvement |
| School 3 | Intervention | Wales | 3 | High | Small | Adequate |
| School 4 | Intervention | Wales | 4 | Low | Large | Good with outstanding features |
| School 5 | Control | England | 1 | Low | Large | Requires improvement |
| School 6 | Control | England | 2 | High | Small | Good |
| School 7 | Control | Wales | 3 | High | Small | Adequate |
| School 8 | Control | Wales | 4 | Low | Large | Good |
