# Supplementary material for: Delivery of a Mental Health First Aid training package and staff peer support service in secondary schools: a process evaluation of uptake and fidelity of the WISE intervention
Source: Trials. 2020 Aug 26;21:745. doi: 10.1186/s13063-020-04682-8 (PMC7448323; doi:10.1186/s13063-020-04682-8)
Supplement: Supplementary file 3 — Additional file 3. Guidelines for peer supporters. Guidelines for peer supporters to set up a peer support service in the school as part of the WISE intervention. [file 13063_2020_4682_MOESM3_ESM.docx]

*
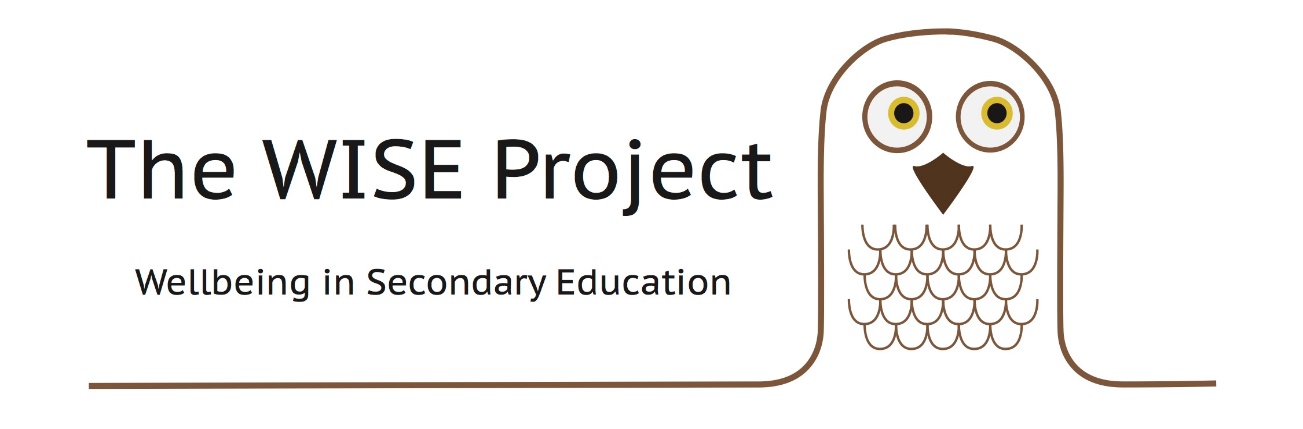
Supplementary material 3. Guidelines for peer supporters*

# **Guidelines for Peer Supporters**

Thank you very much for agreeing to be a peer supporter for your school. The purpose of your role is to form part of a team providing a confidential one-to-one support service to colleagues, offering a listening ear, and, where appropriate, signposting to other services.

Below is a series of suggested guidelines for peer supporters, which we hope will help the service to run as smoothly and effectively as possible.

- We advise you to have a ‘confidentiality policy’ amongst your team, which states that confidentiality of all staff members who approach the service for help will be respected, even between team members. You should include in this policy any exceptional circumstances in which confidentiality may need to be broken, for example if there is a concern that a student may be at risk, and what your procedure should be in this event. You should base such decisions on usual school policy and procedures – we do not expect you to keep things confidential where school policy would dictate otherwise. However, we would expect that in the vast majority of cases, confidentiality can and should be maintained.
- We encourage you to have regular team meetings, to swap ideas and to discuss any problems or concerns arising from a one-to-one session, or about being a peer supporter in general. **Confidentiality of individuals you have supported should be maintained during such discussion.** For this to happen, we suggest that you elect a team member to coordinate these meetings.
- We encourage you to organise yourselves into “buddy pairs”. Each pair would provide ongoing informal support and advice to each other as and when required. Again, **confidentiality of individuals you have supported should be maintained during any discussion between buddies.**
- We suggest that you allow staff members to choose who they would like to approach for a one-to-one peer support session. However, if you find this means one or two of your team have a disproportionately high number of staff approaching them, then you can encourage staff approaching the service towards other team members if they are happy to do this.
- You will need to decide how to advertise the service within the school community. We will be providing leaflets and posters to help with this. You may also want to advertise the service in staff meetings. The information you provide should make your confidentiality procedure clear.
- You will need to agree where you will provide the support requested, and whether you want and are able to have a designated space in school. It is important that support is provided in a place that upholds confidentiality. Other peer supporter teams have found empty classrooms or even walk-in cupboards in which to hold conversations, or have gone for a walk if both staff members are free to do this.
- Please ensure you complete and return the termly logs of support provided to the study team. This information is vital to help us understand the results of the study, and to make future recommendations.

Please get in touch with a member of The WISE Project team if you have any questions or concerns about our research project.

## Contact Details

**XXXXXXX**
