# Supplementary material for: Delivery of a Mental Health First Aid training package and staff peer support service in secondary schools: a process evaluation of uptake and fidelity of the WISE intervention
Source: Trials. 2020 Aug 26;21:745. doi: 10.1186/s13063-020-04682-8 (PMC7448323; doi:10.1186/s13063-020-04682-8)

|  | 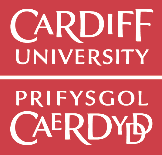 | *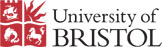* | |  |
| --- | --- | --- | --- | --- |
|  | |  | | |
|  | | |  | |
|  | | |  | |
| 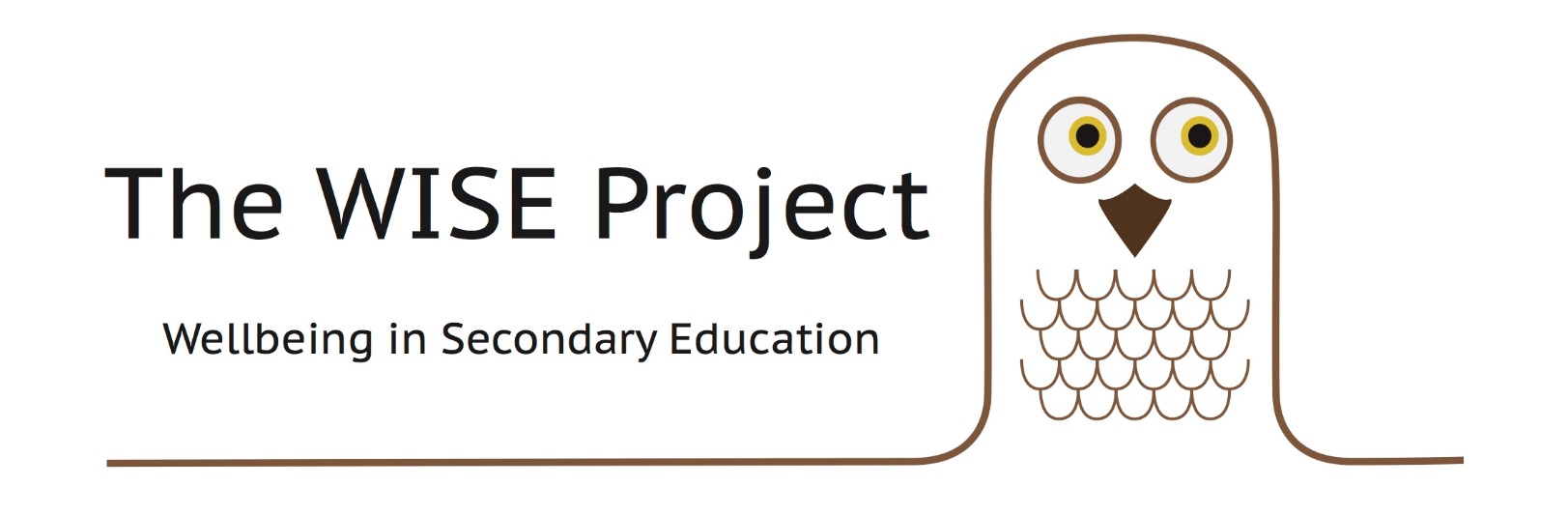 | | | | |
|  | |  | | |
|  | |  | | |
|  | |  | | |
| Observation Guidance  Mental Health First Aid Schools  (1 day session) | | | | |
|  | |  | | |
|  |  | | |  |
|  |  |  | |  |
|  |  |  | |  |
|  |  |  | |  |
|  |  |  | |  |
|  |  |  | |  |

Supplementary material 4. Data collection tools

**Guidance for Assessment of Fidelity, Quality of Delivery, Participant Responsiveness and Adaptation**

**Fidelity**

Assesses if the trainer delivers the session in adherence with the intended learning processes and materials of the session.

***Coverage of Materials***

The extent to which the trainer(s) cover the materials for the sessions. Note that the training is intended to be responsive to the needs of participants so variation will likely occur between trainers and settings in terms of the exact materials delivered. Materials may not necessarily be delivered in the same order across trainers.

| **0 = No Coverage of Material**  Trainer does not cover the materials | **1 = Coverage of Material**  Trainer covers the materials |
| --- | --- |

***Mode of Delivery***

Trainers should deploy a range of slides, group work exercises, film clips, discussion groups, and case studies. Indicate the number of modes of delivery utilised.

| **1 = One mode of delivery**  (e.g. slides) | **2 = Two modes of delivery** | **3= Three modes of delivery**  (e.g. slides, film clips, case studies) | **4 = Four modes of delivery** | **5 = Five modes of delivery**  (e.g. slides, film clips, case studies, discussion groups, role play) |
| --- | --- | --- | --- | --- |

**Quality of Delivery**

Assesses the average quality of the delivery for each of the two instructors. This rating system is consistent with MHFA England’s assessment of instructor quality by participants.

***Knowledge of MHFA***

Assesses: knowledge of course; knowledge of local and national resources

| **1 = Very poor:**  Significant lack of knowledge of the majority of the training course | **2 = Poor:**  Limited knowledge of the training course. Where knowledge is evident it is somewhat superficial | **3 = Neither Poor**  **nor Good:**  Deep knowledge in some areas, but lack of knowledge in others | **4 = Good:**  Deep knowledge across most of the training course | **5 = Very Good:** Extensive, deep knowledge of the training course |
| --- | --- | --- | --- | --- |

***Presentation Skills***

Assesses: establishment of appropriate climate, and includes effective introduction of self, participants and sessions objectives; ensure participants understand aims and expected outcomes; tone and manner; respond to individual needs and learning styles.

| **1 = Very poor:**  Communication and delivery of information very poor. No adaption to individual needs and learning styles | **2 = Poor:**  Poor delivery of core aims and objectives of session. Limited adaption to individual learning styles | **3 = Neither Poor nor Good:**  Good communication of some session objectives but lacked clarify in in others. Accommodation of some learning skills with limited engagement of others | **4 = Good:**  Effective communication of session aims and objectives. Good response to individual learning needs and styles | **5 = Very Good:** Excellent clear communication of session aims and objectives. Excellent engagement and interaction with participants, demonstrating an excellent understanding of differing learning needs |
| --- | --- | --- | --- | --- |

***Facilitation Skills***

Assesses: use of skills and activities to enable and manage learning; balance between group tasks and group process; enable all members to participate; ensure appropriate level and pace of communication; adapt activities to group needs; clear guidance and structure; provide support in appropriate way; demonstrate inclusive practices.

| **1 = Very poor:**  Does not demonstrate facilitation skills | **2 = Poor:**  Limited facilitation skills. May demonstrate some skill but in a limited number of areas | **3 = Neither Poor**  **nor Good:**  Good facilitation skills in some areas but not in other areas | **4 = Good (presentation Skills):**  Good facilitation skills across most of the areas | **5 = Very Good (presentation skills):**  Very good facilitation skills across all areas |
| --- | --- | --- | --- | --- |

***Participant Responsiveness***

Assesses how participants respond to the training course (i.e. the extent to which participants appear interested and engaged with different aspects of the training).

The rating is conducted for the responsiveness of the *whole group*.

Note that a rating of very poor is for individuals who are actively disengaged and may express dissatisfaction with the training content or instructors. A rating of neither poor nor good is for individuals where there is no clear engagement or disengagement. In this case individuals may remain quiet but amenable to taking part in activities when requested to do so, although they require some encouragement.

Note that some individuals may ask a number of questions and challenge the instructor. If this is done in a constructive manner and it appears that individuals are seeking further insight then the responsiveness can be rated as very good. If individuals are challenging in a manner that appears destructive and disruptive then the responsiveness can be rated as poor/very poor.

| **1 = Very poor:**  Participants did not respond well to trainers, course content nor with each other during group work | **2 = Poor:**  Participants showed a reluctance to respond with trainers, course content and with each other during group work | **3 = Neither Poor**  **nor Good:**  Participants were generally indifferent in their response to trainers, course content and each other during group work | **4 = Good:**  Participants responded well with most aspects of the training | **5 = Very Good:**  Participants were enthusiastic to respond and engage with all aspects of the training |
| --- | --- | --- | --- | --- |

***Adaptation and Contextual Observations***

Some elements may be delivered with adaptation. This may be understood as significant changes to a session beyond the intended variation in activities, case-studies and film-clips.

If an adaptation is undertaken, record the detail in the associated adaptation table for each section. If you are not sure if an adaptation has been undertaken make a note of the potential adaptation.

Supplementary notes can be made about general contextual observations.

General contextual points to be considered include:

- How much did the material / case studies relate specifically to schools?
- What particular questions/topics arose?
- How did the instructors interact with attendees? How did they respond to questions?
- How did attendees appear to engage with the course?
- To what extent did attendees draw on their work experiences during discussions etc?
- Were there issues raised that did not appear to be dealt with well?


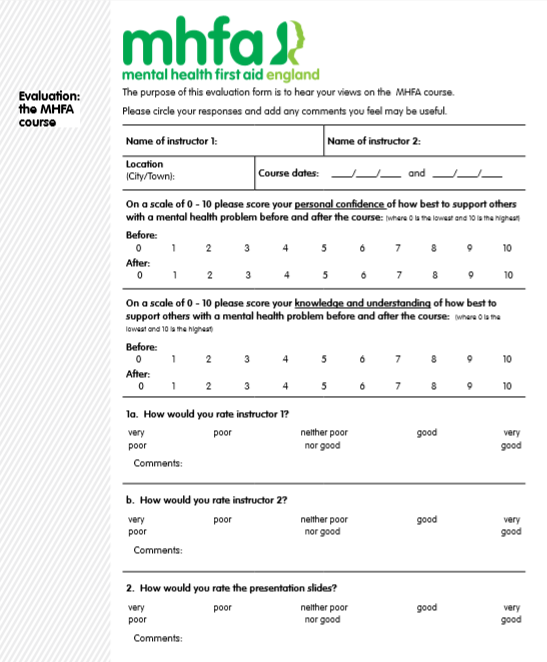


Figure 2. Mental Health First Aid evaluation form


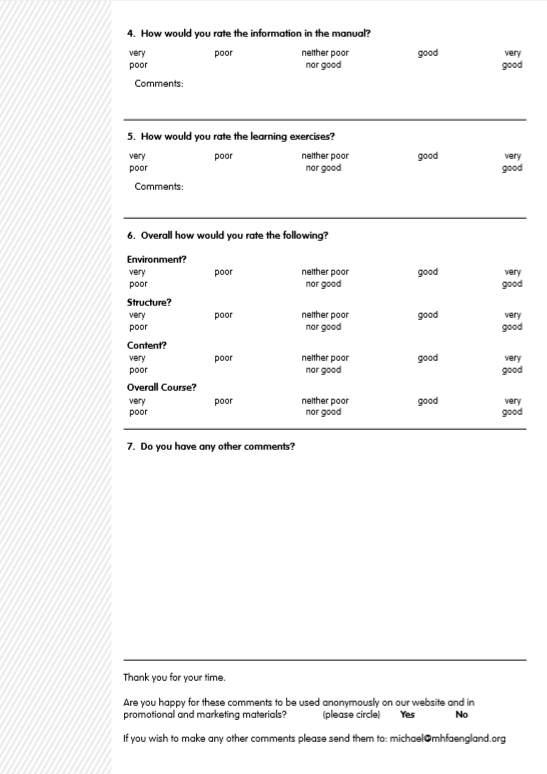


Figure 3. Participant assessed fidelity check lists


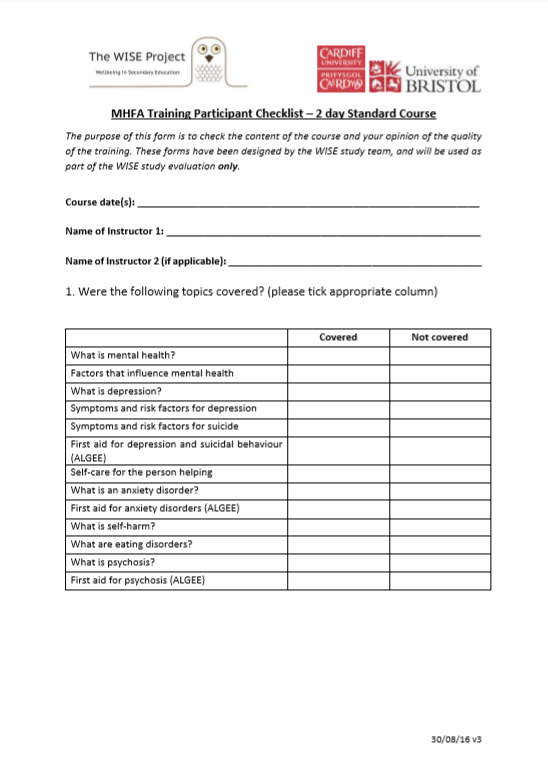

Supplement: Supplementary file 4 — Additional file 4. Data collection tools. Data collection tools: observation schedule template, mental health first aid evaluation forms, participant assessed fidelity check lists. [file 13063_2020_4682_MOESM4_ESM.docx]
