# Supplementary material for: Delivery of a Mental Health First Aid training package and staff peer support service in secondary schools: a process evaluation of uptake and fidelity of the WISE intervention
Source: Trials. 2020 Aug 26;21:745. doi: 10.1186/s13063-020-04682-8 (PMC7448323; doi:10.1186/s13063-020-04682-8)
Supplement: Supplementary file 5 — Additional file 5. Extent of implementation of the WISE intervention at school-level. Table indicating whether domains of fidelity, reach and dosage were achieved by intervention schools. [file 13063_2020_4682_MOESM5_ESM.docx]

| *Supplementary material 5. Extent of implementation of the WISE intervention at school-level* | | | | | | | | | | | | | | |
| --- | --- | --- | --- | --- | --- | --- | --- | --- | --- | --- | --- | --- | --- | --- |
| **School ID** | Dose 1 | Dose 2 | Dose 3 | **Dose total** | Reach 1 | Reach 2 | **Reach total** | Fidelity 1 | Fidelity 2 | Fidelity 3 | Fidelity 4 | Fidelity 5 | **Fidelity total** | **Implementation total** |
| **4V** | No | Yes | No | **1** | No | No | **0** | No | No | No | No | No | **0** | **1** |
| **2A** | No | Yes | No | **1** | No | No | **0** | No | No | No | No | Yes | **1** | **2** |
| **4S** | No | No | No | **0** | No | Yes | **1** | Yes | Yes | No | No | No | **2** | **3** |
| **1D** | Yes | No | No | **1** | No | No | **0** | No | Yes | Yes | Yes | No | **3** | **4** |
| **3X** | Yes | No | No | **1** | No | Yes | **1** | Yes | No | No | No | Yes | **2** | **4** |
| **1I** | Yes | Yes | No | **2** | No | Yes | **1** | Yes | No | No | No | Yes | **2** | **5** |
| **2F** | Yes | Yes | No | **2** | No | No | **0** | Yes | Yes | Yes | No | No | **3** | **5** |
| **1H** | Yes | Yes | Yes | **3** | No | Yes | **1** | No | No | No | Yes | Yes | **2** | **6** |
| **3P** | Yes | Yes | Yes | **3** | Yes | No | **1** | Yes | No | No | No | Yes | **2** | **6** |
| **4Q** | Yes | No | No | **1** | Yes | No | **1** | Yes | Yes | Yes | No | Yes | **4** | **6** |
| **2L** | Yes | Yes | No | **2** | No | Yes | **1** | Yes | Yes | No | Yes | Yes | **4** | **7** |
| **4N** | Yes | Yes | Yes | **3** | No | Yes | **1** | Yes | Yes | Yes | No | Yes | **4** | **8** |

*Key

Dose Q1: At least 8% teachers completed the MHFA for Schools training versus less than 8%

Dose Q2: At least 8% of staff completed the MHFA for adults training and went on to become a peer supporter versus less than 8%

Dosage Q3: At least 8% of staff still acting as peer supporters by time 2 follow up versus less than 8%

Reach Q1: Whether 75% or more of teachers attended the one hour awareness training versus less than 75%

Reach Q2: Higher than study mean for those who select ‘staff peer supporter’ in response to the question ‘if a work related problem was making you stressed or down who would you talk to about it at school?’ in the follow up questionnaires

Fidelity Q1: Whether 100% course attendees indicated that all topics were covered for one and two day courses versus less than 100%

Fidelity Q2: Whether the peer supporters set up a confidentiality policy for the service versus no policy

Fidelity Q3: Whether the peer support service was advertised in three or more ways initially versus advertised in two or fewer ways

Fidelity Q4: Whether the peer support service was re-advertised at the beginning of the 2017-2018 academic year versus not re-advertised

Fidelity Q5: Whether the peer support service has been championed by a member of the senior leadership team versus not championed
